# The antitumoral effect of statins in pancreatic ductal adenocarcinoma: a scoping review

**DOI:** 10.3389/fphar.2026.1815366

**Published:** 2026-04-29

**Authors:** Alexandru-Ioan Pîntea, Adina Emilia Croitoru, Cristina Elena Dinu-Pîrvu, Simona Olimpia Dima

**Affiliations:** 1 Fundeni Clinical Institute, Bucharest, Romania; 2 Carol Davila University of Medicine and Pharmacy, Bucharest, Romania; 3 Innovative Therapeutic Structures Research and Development Centre (InnoTher), “Carol Davila” University of Medicine and Pharmacy, Bucharest, Romania; 4 Center of Excellence in Translational Medicine (CEMT), Fundeni Clinical Institute, Bucharest, Romania

**Keywords:** cholesterol biosynthesis, lipid metabolism, pancreatic ductal adenocarcinoma, PDAC, statins

## Abstract

**Introduction:**

Pancreatic ductal adenocarcinoma (PDAC) remains one of the most aggressive and invasive malignancies, with limited response to the currently available systemic therapy options. Having a 5-year survival of 12%, there is a serious need for novel therapeutic options, including drug repurposing for the treatment of PDAC. In recent years, 3-hydroxy-3-methylglutaryl-coenzyme A reductase (HMGCR) inhibitors, also known as statins, have been identified as potential candidates for PDAC therapy repurposing.

**Methods:**

We have performed a scoping literature review to summarize the extent of knowledge on statins’ effects on PDAC, both in clinical studies and preclinical models, and to explain the mechanisms underlying statins’ antiproliferative effect on PDAC. We have followed the PRISMA Extension for Scoping Reviews (PRISMA-ScR) guidelines. We have searched PubMed and Web of Science for original articles published between January 2021 and January 2026.

**Results:**

Our review systematically linked the clinical outcomes, molecular mechanism, and tumor microenvironment in PDAC-statin research. Clinical studies of the correlation between statin treatment and PDAC incidence yielded mixed results. Among the mechanisms we have summarized are disruption of the plasma membrane lipid rafts, reduction of protein prenylation, increase of the tumor immunogenicity, reduction of signaling, both RAS-mediated and by alternative pathways, and disruption of mitochondrial respiration. Statins also showed synergistic effects with chemotherapeutic agents including gemcitabine, 5-fluorouracil, and oxaliplatin in preclinical models.

**Discussion:**

Despite promising preclinical data, the clinical evidence remains limited to observational studies and a single phase 2 trial. Randomized controlled trials with biomarker-driven patient selection are needed to establish the role of statins as adjuvant therapy in PDAC.

## 1 Introduction

Pancreatic ductal adenocarcinoma (PDAC) remains one of the most aggressive cancers, with a median 5-year survival of 12% (Siegel et al., 2023), and very limited treatment options. PDAC is estimated to become the second-highest mortality cancer in several Western countries within the next decade, emphasizing the necessity for novel therapeutic approaches (Rahib et al., 2014). In the adjuvant setting, among the common systemic therapy regimens are mFOLFIRINOX (a chemotherapy triplet containing 5-fluorouracil, irinotecan and oxaliplatin) and the combination of gemcitabine and capecitabine, the former having yielded a median overall survival (OS) of 53.5 months in the PRODIGE 24 trial (Conroy et al., 2022), with a 5-year survival of 43.2%, while the latter a median OS of 29.5 months according to the ESPAC-4 trial (Palmer et al., 2025), with a 5-year survival of 32%. In the advanced/metastatic disease, the median OS of the patients treated with mFOLFIRINOX is 11.1 months (Conroy et al., 2011), while the patients treated with other systemic therapy regimens, such as gemcitabine + nab-paclitaxel have a median OS of 8.5 months according to the MPACT trial (Hoff et al., 2013). A newer regimen, NALIRIFOX (a chemotherapy triplet of 5-fluorouracil, liposomal irinotecan, and oxaliplatin) yielded similar results: a median OS of 11.1 months in the NAPOLI-3 trial (Wainberg et al., 2023).

Apart from chemotherapy, targeted therapies show promise in the treatment of PDAC, but with modest results to date. Approximately 90% of PDAC cases harbor a Kirsten rat sarcoma (KRAS) mutation (Prior et al., 2012). The development of KRAS G12C inhibitors such as sotorasib and adagrasib marked a meaningful advance in precision oncology, but their relevance in pancreatic ductal adenocarcinoma remains limited because KRAS G12C is present in only about 1%–2% of PDAC cases (Luo, 2021). Drugs with broader activity, including pan-KRAS and KRAS G12D inhibitors, are now in development and may prove more useful in this disease (Hallin et al., 2022). In the subgroup of BRCA-mutated PDAC, which accounts for roughly 5%–7% of patients, olaparib has already shown benefit as maintenance treatment after platinum-based chemotherapy (Golan et al., 2019). By contrast, immune checkpoint blockade has produced little benefit in PDAC overall, with meaningful responses largely restricted to the microsatellite instability-high/mismatch repair deficient (MSI-H/dMMR) patients, who represent 1%–2% of PDAC cases (Luchini et al., 2021; Marabelle et al., 2025). Because the therapeutic options in PDAC are very limited, drug repurposing strategies that can complement existing therapies are needed.

The PDAC cells’ reliance on lipid (especially cholesterol metabolism) is among the many resistance mechanisms of the PDAC (Guillaumond et al., 2015), thus it has been hypothesized that cholesterol-lowering drugs such as 3-hydroxy-3-methylglutaryl-coenzyme A reductase (HMGCR) inhibitors (statins) may play a role in the systemic therapy of PDAC (Tamburrino et al., 2020). The mevalonate pathway byproducts, farnesyl pyrophosphate (FPP) and geranylgeranyl pyrophosphate (GGPP), determine the KRAS protein prenylation which in turn determines the KRAS anchoring to the cell membrane, facilitating the KRAS-mediated cell signaling (Longo et al., 2020). Thus, it has been hypothesized that mevalonate pathway inhibition by statins could reduce the KRAS-mediated pro-survival cell signaling, giving a rationale for the investigation of statins as potential adjuvant therapy in PDAC (Tamburrino et al., 2020).

Earlier reviews and meta-analyses, especially Tamburrino et al. (2020), are generally focused on the clinical impact of statins on survival. In the past 5 years, however, the field has expanded considerably on the preclinical side. Newer studies have included patient-derived organoid (PDO) models, examined mechanisms of immune regulation, explored interactions within the tumor microenvironment, and tested a range of combination approaches. Furthermore, 2025 brought the publication of the first reported clinical trial results evaluating statins in patients with PDAC (NCT06241352) (Li et al., 2025). This scoping review aims to map and synthesize the extent and nature of recent evidence (2021–2026) on statins’ effects on PDAC, both in clinical and preclinical settings, and to identify gaps in the current knowledge base.

## 2 Materials and methods

This scoping review was conducted in accordance with the PRISMA Extension for Scoping Reviews (PRISMA-ScR) (Tricco et al., 2018). The review question was made using the Population, Concept, Context (PCC) framework: Population – PDAC patients, cancer-free subjects, or human PDAC preclinical models; Concept – statin treatment and its antitumoral effects; Context – clinical studies (observational and interventional) and preclinical models (cell cultures, organoids, xenografts). A study protocol was written in advance.

We have performed a systematic literature search in PubMed and Web of Science databases of articles published 5 years before the 12th of January 2026. This timeframe was chosen to map the most recent evidence, building upon the comprehensive meta-analysis on the statins’ impact on PDAC patients’ survival (Tamburrino et al., 2020). This focused window is justified by the rapid expansion of mechanistic preclinical work and the emergence of the first clinical trial data involving statins in PDAC, both of which postdate 2020. We have searched for original studies of either PDAC patients or cancer-free subjects or preclinical models, such as cell cultures of human PDAC cells, human PDAC patient-derived organoids, or PDAC xenografts implanted in animals, that were treated with statins and compared with patients, respectively, and preclinical models that were not treated with statins. In the clinical studies, the outcomes were overall survival in pancreatic cancer patients, and pancreatic cancer incidence in the initially cancer-free subjects, and the rate of proliferation or cell viability in the preclinical models.

The search query on PubMed was (“PDAC” OR “pancreatic ductal adenocarcinoma” OR “pancreatic cancer”) AND (“statins” OR “statin” OR “HMG-CoA-reductase inhibitors” OR “atorvastatin” OR “lovastatin” OR “simvastatin” OR “rosuvastatin”). The search query on Web of Science was: TS = ((PDAC OR pancreatic ductal adenocarcinoma OR pancreatic cancer) AND (statins OR statin OR HMG-CoA-reductase inhibitors OR atorvastatin OR lovastatin OR simvastatin OR rosuvastatin)).

The inclusion criteria: original articles, PDAC patients or human PDAC preclinical models, statin treatment. Exclusion criteria: non-original article (e.g., reviews, meta-analyses, conference abstracts), non-peer-reviewed articles, non-PDAC, non-human PDAC cells preclinical models, no statin intervention, articles older than 5 years, non-English language articles.

After the search was completed, duplicates were removed, and titles and abstracts were screened. Subsequently, the full-text reports were retrieved, and a screening of the full-text articles was conducted. The screening process was carried out independently by two reviewers. A PRISMA flow chart for the selection of the studies was created.

Data extraction used a charting form, developed by three reviewers (A.I.P, A.E.C., and S.D.) that included the following variables: author(s), year, study design, population/model, sample size, statin type and dose, exposure timing and duration, comparator, outcomes, key findings, and study limitations. Data charting was performed independently by two reviewers (A.I.P. and S.D.), with discrepancies resolved through discussion and consensus.

Due to expected heterogeneity across clinical and preclinical evidence, we performed a narrative synthesis of the selected studies. The absence of a third database is acknowledged as a limitation of the search process.

## 3 Results

### 3.1 Data extraction

**FIGURE 1:**
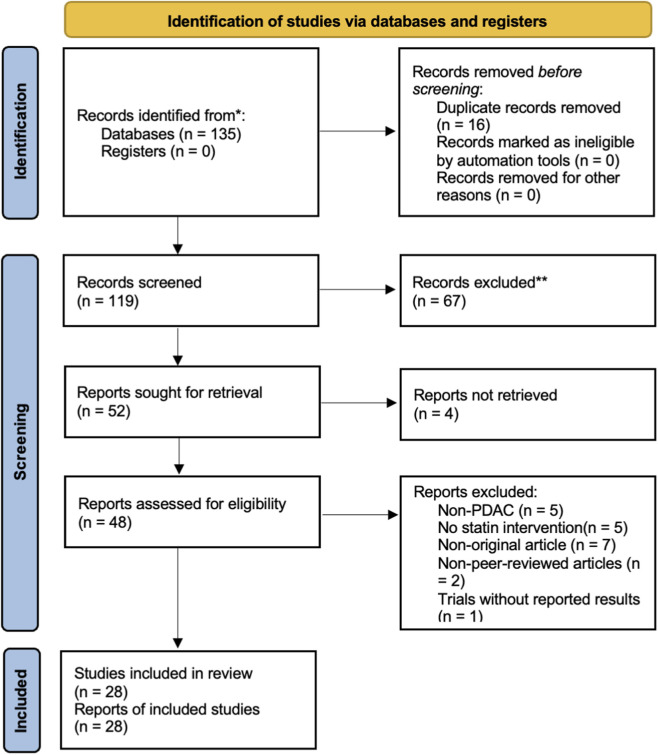
The PRISMA flow diagram.

Out of 135 records retrieved after the search, 16 duplicates were removed. At the titles and abstract screening, 67 records were removed. There were 4 full-text reports we were unable to retrieve. Of the full-text reports, 20 were excluded, and 28 reports were included for the review. The process is summarized in a PRISMA flow diagram in Figure 1.

### 3.2 Clinical studies assessing statin use in PDAC patients

In the past 5 years, new results of retrospective and prospective studies of statin use, mainly in Asian populations, emerged, having as endpoints cancer incidence (including PDAC incidence) in subjects not yet diagnosed with cancer, or OS, disease-free survival (DFS)/relapse-free survival (RFS) in cancer patients (including PDAC patients) (Guo et al., 2025; Joliat et al., 2023; Okita et al., 2024; Stoer et al., 2021; Uemura et al., 2023; Xu et al., 2024; Yeh et al., 2021).

Evidence from large population-based cohorts demonstrates mixed results regarding the relationship between statin use and PDAC incidence or survival outcome, with some cohorts showing variable protective effects and others showing a survival benefit of statin exposure, as shown in Table 1 (Guo et al., 2025; Joliat et al., 2023; Okita et al., 2024; Stoer et al., 2021; Uemura et al., 2023; Xu et al., 2024; Yeh et al., 2021).

**TABLE 1:** Results from clinical studies of statin use and their impact on PDAC incidence, DFS, RFS, OS and prognosis.

Study	Study design and population	Country/Region	Sample size	Endpoints	Statin type	Statin exposure definition	Exposure timing	Results (HR, 95% CI)	Key adjustment variables	Potential sources of bias	Dose-Response/Duration analysis	Strength of evidence	Conclusions
PDAC incidence studies													
Okita et al. (2024)	Prospective cohort, general population 10-year follow-up	Japan	67,768 individuals	PDAC incidence	Any statin (anti-cholesterol drugs)	Prescription records <5 years vs. > 5 years vs. no use	Pre-diagnosis (primary prevention)	Total PDAC: 335 cases <5 years: HR 0.88 (95% CI 0.53–1.45) >5 years: HR 1.77 (95% CI 1.16–2.70) p = 0.04	Age, sex, BMI, diabetes, smoking alcohol, physical activity	Protopathic bias (early PDAC symptoms may prompt statin Rx) surveillance bias confounding by metabolic syndrome	Yes: duration categories (<5 years, >5 years); p-trend = 0.04	Low (observational single cohort Potential bias)	Long-term statin use (>5 years) associated with increased PDAC risk
Xu et al. (2024)	Observational real-world cohort, statin initiators matched to non-users (target trial emulation)	Hong Kong China	205,177 individuals (118,914 statin initiators)	PDAC incidence	Any statin	New statin initiation 2009–2011; matched to non-users	Pre-diagnosis (primary prevention)	Total PDAC: 329 cases HR 0.98 (95% CI 0.61–1.60)	Propensity score matching on age sex, comorbidities medications	Residual confounding; healthy user bias Hong Kong–specific population short follow-up for some participants	Not reported	Moderate (target trial emulation design large sample)	No impact of statin use on PDAC incidence
Yeh et al. (2021)	Retrospective cohort of ILD/pulmonary fibrosis patients	Taiwan	53,862 ILD and pulmonary fibrosis patients	PDAC incidence	Any statin	Prescription records; statin use vs. no statin use time-dependent exposure	Pre-diagnosis (primary prevention)	Adjusted HR 0.42 (95% CI 0.29–0.63)	Age, sex, comorbidities medications Propensity score	Confounding by indication (ILD/PF Population) non-generalizable to general population time-dependent exposure modeling	Time-dependent analysis performed	Moderate (large cohort, but specific Population)	Statin use associated with reduced PDAC incidence
Survival studies (OS, DFS, RFS)													
Guo et al. (2025)	Retrospective cohort of cancer patients	United States	4,538 PDAC patients	OS	Any statin (post-diagnosis)	Post-diagnosis statin use duration categories no use (ref) <1 year, 1–2 years 2–3 years, >3 years	Post-diagnosis	OS HR (95% CI) <1 year: 0.77 (0.62–0.96) 1–2 years: 0.53 (0.34–0.83) 2–3 years: 0.27 (0.10–0.73) >3 years: 0.52 (0.25–1.08) p-trend = 0.04	Age, sex, race, cancer stage treatment comorbidities statin pre-diagnosis	Immortal time bias; confounding by indication; healthy user effect retrospective design	Yes: 4 duration categories significant p-trend = 0.04 Peak at 2–3 years	Moderate (large cohort, dose-response but retrospective)	Post-diagnosis statin use associated with improved OS. Increasing benefit with longer duration (up to 3-year use)
Stoer et al. (2021)	Retrospective European (Norwegian) cohort	Norway	2,614 pancreatic cancer patients	OS	Lipophilic and hydrophilic (compared)	Prescription registries statin use vs. non-use	Pre- and post-diagnosis (registry-based)	OS HR 0.86 (95% CI 0.76–0.97) Lipophilic vs. hydrophilic no significant difference	Age, sex, cancer stage comorbidities other medications	Immortal time bias; confounding by indication retrospective no dose data	Lipophilic vs. hydrophilic comparison non-significant difference	Moderate (nationwide registry large sample)	Statin use associated with improved OS no difference between lipophilic and hydrophilic
Uemura et al. (2023)	Retrospective surgical cohort	Japan	47 resected PDAC patients	OS, RFS	Any statin (perioperative)	Perioperative statin use (medical records)	Perioperative	OS HR 0.61 (95% CI 0.37–0.96) p = 0.024 Median RFS 27 vs. 14 months, p = 0.049	Age, sex, stage, adjuvant chemotherapy CA19-9, tumor size, node status	Very small sample (n = 47); selection bias; retrospective non-randomized	Not reported	Low (very small sample single center retrospective)	Peri-operative statin use associated with improved OS and RFS
Joliat et al., 2023	Retrospective cohort, upfront pancreato-duodenectomy 3 centers	Switzerland France United States	496 PDAC patients	OS, DFS	Any statin (preoperative)	Preoperative statin medication (medical records)	Preoperative	No statistically significant differences in OS or DFS	Age, sex, BMI, diabetes, stage,CA19-9 adjuvant chemotherapy R status	Retrospective; multicentric heterogeneity no dose/duration data; potential underpowering	Not reported	Moderate (multicentric, reasonable n but retrospective)	No survival benefit associated with preoperative statin use
Clinical trials													
Li et al. (2025) (NCT06241352)	Single-arm phase II clinical trial	China	42 locally advanced/metastatic PDAC	CA19-9 response PFS	Atorvastatin 80 mg/day	Atorvastatin added to ongoing chemo at CA19-9/CEA Plateau	During chemotherapy (at marker plateau)	≥20% CA19-9 reduction in 70.3%; median PFS 8.05 months	N/A (single-arm; no comparator adjustment)	No comparator arm; small sample (n = 42) surrogate endpoint (CA19-9); concurrent chemo confounding single-arm design	N/A (single dose regimen)	Low (single-arm, no comparator surrogate endpoint small n)	Statins show biological activity signal of anti-tumoral effect

HR, hazard ratio; CI, confidence interval; OS, overall survival; DFS, disease-free survival; RFS, relapse-free survival; PFS, progression-free survival; NR, not reported.

Okita et al., in a prospective study of a Japanese cohort with a 10-year follow-up period (Okita et al., 2024), divided subjects into three statin use categories: no statin use (the reference), <5 years statin use, and >5 years statin use (Okita et al., 2024). The PDAC incidence rate in the >5 years of use was significantly higher with a hazard ratio (HR) of 1.77, and 95% confidence interval (CI) of 1.16–2.70, p = 0.04, which would indicate the long-term statin use as a PDAC development risk factor. However, this finding should be interpreted with caution given the potential for protopathic bias (early PDAC symptoms such as new-onset diabetes may have prompted statin initiation) and surveillance bias (long-term statin users undergo more frequent medical monitoring). Similar results have been obtained in an observational cohort analysis using real-world evidence in a Hong Kong cohort (Xu et al., 2024) of 118,914 individuals who started statin treatment between 2009 and 2011 and were matched to non-statin using subjects. This study didn’t report any impact of statin use on PDAC incidence (HR 0.98, 95% CI 0.61–1.60). On the other hand, another study, this time of Taiwanese interstitial lung disease (ILD) and pulmonary fibrosis patients, reported a benefit of statin use in terms of PDAC incidence (adjusted HR 0.42, 95% CI 0.29–0.63) (Yeh et al., 2021).

The contradictory findings across these three studies (Okita et al., 2024; Xu et al., 2024; Yeh et al., 2021) likely reflect differences in study populations (general population vs. ILD/pulmonary fibrosis patients), geographic and ethnic factors, confounding by indication (statin users inherently differ from non-users due to cardiovascular comorbidities), and heterogeneous definitions of statin exposure.

Beyond risk reduction, the impact of statins on cancer survival has also been examined. In terms of OS and DFS/RFS, there are a few encouraging reports, with generally more consistent, though not uniform, positive signals (Guo et al., 2025; Stoer et al., 2021; Uemura et al., 2023; Joliat et al., 2023).

In resectable disease, the evidence consistently reveals that perioperative statin use was associated with improved overall survival. Uemura et al. compared the OS and RFS outcomes of 47 resected PDAC patients with perioperative statin use with those of never-treated with statins resected PDAC patients, reporting statistically significant differences, favoring the statin intake group (Uemura et al., 2023).

On the other hand, in another retrospective study conducted by Joliat et al., the impact of preoperative statin administration to 496 PDAC patients referred to upfront pancreatoduodenectomy in three centers (Joliat et al., 2023) was reported. This study offers no data about postoperative statin exposure of the PDAC patients. No statistically significant differences in OS and DFS between the patients treated with statins and those never treated with statins were reported (Joliat et al., 2023).

In a retrospective study of cancer patients, of which 4,538 were PDAC patients (Guo et al., 2025), Guo et al. reported a benefit on OS for the PDAC patients treated with statins after diagnosis compared with the PDAC patients never treated with statins. In the same study, the impact on survival was analyzed on statin use duration categories: <1 year, 1–2 years, 2–3 years, >3 years, with higher benefits on OS in the 1–2 years category compared to <1 category, and respectively 2–3 years category compared to 1–2 years category, with a p_trend_ value of 0.04 (Guo et al., 2025). Similarly, in a retrospective study of a European population (Stoer et al., 2021), Stoer et al., in a nationwide Norwegian study, reported a better OS in the statin-treated PDAC patients compared to the PDAC patients not treated with statins (HR 0.86, 95% CI 0.76–0.97). The study compared the effect of lipophilic statins with the effect of the hydrophilic statins, with no statistically significant difference (Stoer et al., 2021).

NCT06241352 (Li et al., 2025) is, to the best of our knowledge, the only clinical trial with reported results involving statins administration in PDAC patients to assess statins’ antitumoral effects. NCT06241352 is a single-arm phase 2 clinical trial, it had 42 locally advanced or metastatic pancreatic cancer patients enrolled who had reached a carbohydrate antigen 19–9 (CA19-9) or carcinoembryonic antigen (CEA) plateau during systemic chemotherapy, which is considered a warning sign for chemotherapy resistance. The trial’s primary endpoint was a decrease of 20% of CA19-9 within a month of statin treatment. Patients received atorvastatin 80 mg/day, daily, alongside their usual chemotherapy regimens. The median progression-free survival was 8.05 months. The primary endpoint was reached in 70.3% of the participants (Li et al., 2025).

Several limitations of NCT06241352 should be noted. The single-arm trial design does not allow comparison with patients not receiving statins. Furthermore, the trial does not measure either OS or overall response rate, but instead uses a surrogate, the decrease in CA19-9. However, this trial’s results are encouraging and warrant further clinical research on statins’ effect in PDAC patients.

### 3.3 Preclinical data in PDAC models

The mixed and largely inconclusive clinical evidence highlights the need to understand the molecular mechanisms through which statins exert their effects on PDAC. Preclinical studies over the past 5 years have identified multiple, interconnected antitumoral pathways, which we have organized thematically below. The half-maximal inhibitory concentration (IC50) values from preclinical studies that report them and are included in this scoping review (Duranti et al., 2025; Gyoten et al., 2023; Ou et al., 2022; Rimpelová et al., 2021; Xing et al., 2024) are summarized in Table 2. Where specific IC50 values were not calculated by the original authors, we report the concentration ranges.

**TABLE 2:** IC50 data from the included preclinical studies.

Study	Statin	Cell Line/Model	IC50 (µM)	Exposure duration	Endpoint	Target/Mechanism
Rimpelová et al., 2021	Cerivastatin	MIA PaCa-2	10	24 h	Cell viability	Transcriptomic profiling
	Simvastatin		12			
	Lovastatin		13			
	Pitavastatin		20			
	Fluvastatin		26			
	Atorvastatin		27			
	Pravastatin		29			
	Rosuvastatin		36			
Gyoten et al., 2023	Lovastatin	MIA PaCa-2, PANC-1	0.1–1 (MIA PaCa-2)	48 h	Cell viability, apoptosis, invasion, sphere formation, gemcitabine resistance, mitochondrial oxidative stress	Lipid rafts, EGFR-Akt, mitochondria
	Simvastatin		1–10 (MIA PaCa-2)		Cell viability	
	Fluvastatin		10–100 (MIA PaCa-2)			
	Rosuvastatin		100–1,000 (MIA PaCa-2)			
Duranti et al., 2025	Simvastatin	MIA PaCa-2, PANC-1	4.47 ± 0.48 (PANC-1) 4.16 ± 1.02 (MIA PaCa-2)	24 h (viability) 90 min (motility)	Cell viability, proliferation, spheroids, migration combination synergy with scDb-hERG1-β1	hERG1/β1 integrin, lipid rafts, PI3K/Akt combination index with scDb: 0.27 ± 0.01 (PANC-1) 0.37 ± 0.03 (MIA PaCa-2)
	Lovastatin		2.79 ± 0.84 (PANC-1) 2.07 ± 1.01 (MIA PaCa-2)	24 h (viability) 90 min (motility)	Cell viability, migration	hERG1/β1 integrin, lipid rafts combination with scDb-hERG1-β1 increased effect formal combination index NR
	Fluvastatin		2.47 ± 1.06 (PANC-1) 3.45 ± 1.05 (MIA PaCa-2)	24 h (viability) 90 min (motility)	Cell viability, migration	hERG1/β1 integrin, lipid rafts combination with scDb-hERG1-β1 increased effect formal combination index NR
	Atorvastatin		1.81 ± 1.12 (PANC-1) 3.13 ± 1.13 (MIA PaCa-2)	24 h (viability) 90 min (motility)	Cell viability, migration combination synergy with scDb-hERG1-β1	hERG1/β1 integrin, lipid rafts, PI3K/Akt combination index with scDb: 0.61 ± 0.02 (PANC-1) 0.63 ± 0.01 (MIA PaCa-2)
Ou et al., 2022	DZ-SIM (simvastatin conjugate)	MIA PaCa-2, BxPC-3, CS-P-124, CS-P-2	6.9 (MIA PaCa-2); 7.2 (BxPC-3), 9.5 (CS-P-124), 12.3 (C-S-P-2)	24 h	Cell viability, colony formation	Mitochondrial accumulation
Xing et al., 2024	Simvastatin	MIA PaCa-2, PANC-1, Panc02	8.524 (PANC-1) 4.533 (MIA PaCa-2) 5.404 (Panc02)	72 h (viability)	Cell viability, GGPP rescue, pyroptosis, organoids KPC allografts/survival 5-FU combination	Mevalonate pathway (HMGCR/GGPP) Simvastatin showed a synergistic effect with 5-FU in all three pancreatic cancer cell lines by Loewe analysis. The strongest synergy was observed at 1 μM 5-FU + 5 μM simvastatin in PANC-1 (synergy score 13.047), 1 μM 5-FU + 0.5 μM simvastatin in Panc02 (synergy score 11.213), and 1 μM 5-FU + 1 μM simvastatin in MIA PaCa-2 (synergy score 12.682). GGPP supplementation reduced 5-FU sensitivity, supporting involvement of the mevalonate pathway

NR, not reported.

#### 3.3.1 Mevalonate pathway regulation and cholesterol metabolism

While statins are well-established inhibitors of the mevalonate pathway, which is critical for cholesterol biosynthesis and the production of inflammatory mediators, their broader impact on cellular processes relevant to cancer biology remains incompletely understood (Tamburrino et al., 2020). The mevalonate pathway is an important metabolic pathway that converts mevalonate into sterol isoprenoids, such as cholesterol, and into several hydrophobic molecules, nonsterol isoprenoids (Buhaescu and Izzedine, 2007). The mevalonate pathway is centrally regulated at the transcriptional level by sterol regulatory element-binding proteins (SREBPs) (Buhaescu and Izzedine, 2007).

Preclinical studies suggest that the mevalonate pathway inhibition may represent a strategy for enhancing therapeutic response in PDAC. Moreover, inhibition of the mevalonate pathway exerts a strong impact on several oncogenic signaling pathways (Mantha et al., 2005).

Recent research highlights that the Kinesin Family Member 11 (KIF11)/mevalonate axis could serve as a new predictive biomarker and a potential therapeutic target for the progression and treatment of PDAC (Gu et al., 2022). KIF11 is an essential component of the bipolar division. Gu et al. determined that KIF11 is highly expressed in PDAC cells and upregulates and stabilizes SREBP2, which in turn upregulates the mevalonate pathway, leading to PDAC cell proliferation. Further, using PDAC cell xenografts implanted in mice, it was demonstrated that atorvastatin has efficiently suppressed PDAC KIF11-high tumor growth (Gu et al., 2022).

Transcription factor CP2 (TFCP2) is upregulated in KRAS mutant PDAC, is an anti-senescence factor, and cooperates with SREBP2, together with synergistically activating effect on HMGCR. By treating HPAC cells with statins, it was discovered that statins block the TFCP2-mediated senescence escaping (Zhang et al., 2021).

#### 3.3.2 Prenylation-dependent oncogenic signaling

KRAS mutations may influence cholesterol metabolism through additional, SREBP-independent mechanisms. Nam et al. demonstrated that simvastatin has a better antiproliferative effect in KRAS mutant cancers compared with KRAS wild-type by comparing the effect of simvastatin on multiple murine and human colorectal cancer cell lines with different mutational profiles (Nam et al., 2021). Interestingly, the only KRAS mutant lines without a statistically significant better response to simvastatin were those that also had BRAF mutations. The simvastatin effect in the CT26 murine KRASG12D cell line was reversed by the addition of mevalonate pathway metabolites involved in prenylation, FPP, and GGPP, supporting the idea that simvastatin acts by blocking KRAS protein prenylation. Nam et al. also demonstrated that statin-mediated inhibition of RAS prenylation activated endoplasmic reticulum (ER) stress, which in turn enhanced the immunogenicity of KRAS mutant cancer cells (Nam et al., 2021).

Nam et al. also performed experiments on the KIC (p48Cre; LSLKrasG12D; Cdkn2af/f) mice – a genetically engineered mice model (GEMM) where PDAC occurs *de novo*. The mice were divided into 4 groups: treated with simvastatin and oxaliplatin, treated with simvastatin, treated with oxaliplatin, and control. The first group had statistically significantly better survival outcomes than the other groups. The simvastatin + oxaliplatin group also had infiltrates with more CD8^+^ T-cells than the other groups (Nam et al., 2021). This chemopreventive effect demonstrates that mevalonate pathway inhibition can block KRAS-driven tumorigenesis at early stages, suggesting potential applications in high-risk populations.

#### 3.3.3 Lipid raft disruption and receptor complex disassembly

A convergent mechanism identified across multiple studies is the disruption of plasma membrane lipid rafts by statins through intracellular cholesterol depletion (Duranti et al., 2025; Gyoten et al., 2023).

It was determined that a complex formed of the hERG1 potassium channel and β1 integrin is preferentially local in the cell membrane lipid rafts (Duranti et al., 2025). The disruption of hERG1/β1 complex or lipid rafts by the bispecific antibody scDb-hERG1-β1 antibody and methyl-β-cyclodextrin (MβCD), respectively, in PDAC cell lines, decreases cell signaling by mechanisms such as reduction of the p85 subunit of phosphoinositide 3-kinase (PI3K), decreasing levels of phosphatidylinositol-3,4,5-triphosphate (PIP3), increasing phosphatidylinositol-4,5-bisphosphate (PIP2), and reducing Akt phosphorylation. The treatment of the PDAC cell lines with statins: simvastatin, lovastatin, fluvastatin, atorvastatin disrupted the lipid rafts and the hERG1/β1 complex by reducing intracellular cholesterol, also significantly reducing cell viability in 24 h after treatment. Further, the effect of the combination of statins with gemcitabine and oxaliplatin was tested on cell lines, where they showed a synergistic effect (Duranti et al., 2025).

Gyoten et al. observed, in three gemcitabine-resistant PDAC cell lines, an increased activity of the epidermal growth factor receptor (EGFR)-Akt axis, which in turn increased the binding of B-cell lymphoma 2 (BCL2)-Bcl-2-associated X protein (BAX), thus suppressing apoptosis. This effect was countered by the lovastatin treatment, by disrupting the lipid rafts in the cell and mitochondria membranes, and by increasing mitochondrial oxidative stress (Gyoten et al., 2023).

#### 3.3.4 Cell cycle regulation, apoptosis, and antiproliferative effects

Uemura et al. determined that statins have antiproliferative properties, in decreasing order of potency: simvastatin, fluvastatin, atorvastatin, rosuvastatin, pravastatin. Simvastatin activates the C-Jun N-terminal kinase (JNK) pathway, thus downregulating Yes-associated protein (YAP)/transcriptional coactivator with PDZ-binding motif (TAZ), having antiproliferative effect. By downregulating TAZ, simvastatin suppresses the expression of PD-L1, as demonstrated in MIA PaCa-2, PANC-1, PK-8, and AsPC-1 cell cultures. Lipophilic statins showed a stronger antiproliferative effect than hydrophilic statins. Simvastatin dose-dependently decreased YAP/TAZ in MIA PaCa-2 cells, but not in PK-8 cells. Simvastatin dose-dependently induced apoptosis in MIA PaCa-2 cells, highlighted by the cleaved caspase-3 expression. These discoveries point out that statins have pro-apoptotic effects on PDAC cells by inhibiting YAP/TAZ expression and activating JNK (Uemura et al., 2023).

Simvastatin’s antiproliferative effects have been confirmed in two studies on human PDAC cell lines. Chen et al. demonstrated that simvastatin decreased the viability of PDAC cells in cell lines such as MIA PaCa-2 cells, PANC-1 cells, and BxPC-3 by G0 cell cycle arrest (Chen et al., 2022). The simvastatin-induced growth inhibition persisted after simvastatin-exposed cells were implanted in mice: subcutaneous, intraperitoneal and intravenous (Chen et al., 2022). In another study, it was established that cerivastatin, pitavastatin, and simvastatin had the most significant transcriptomic impact on MIA PaCa-2 PDAC cell line, producing growth arrest and cytoskeletal remodeling, compared to lovastatin, fluvastatin, atorvastatin, pravastatin, and rosuvastatin (Rimpelová et al., 2021).

Apart from direct growth inhibition, statins can impair the tridimensional organization of PDAC cells. An *in vitro* study on the MIA PaCa-2 line and adipose-derived mesenchymal stem cells (ADMSC) spheroids demonstrated the antiproliferative effects of statins on PDAC cells. On the other hand, the statin concentration that affects PDAC cell viability does not have the same effect on ADMSC spheroids. Statins, however, have disorganized the tridimensional spheroid structures (Gbelcová et al., 2024).

#### 3.3.5 Mitochondrial stress and dysfunction

Several studies have identified mitochondrial dysfunction as a mechanism contributing to statins’ antitumoral effect in PDAC. Ou et al. developed a new drug resulted from conjugating near-infrared organic heptamethine carbocyanine dye with simvastatin (DZ-SIM), which was tested against the components of the conjugate in human PDAC cell lines MIA PaCa-2 and BxPC-3 where it significantly reduced cell viability. Also, *in vitro*, DZ-SIM demonstrated dose-dependent inhibition of colony forming and cell migration. It was demonstrated that the anticancer effect is due to the DZ-SIM accumulation in the mitochondria and the subsequent mitochondrial dysfunction (Ou et al., 2022).

Desai et al. demonstrated that the combination of atorvastatin and biochanin A synergistically decreased the viability of PDAC cells in PANC-1, AsPC-1, MIA PaCa-2 lines, the underlying mechanism being the inhibition of mitochondrial respiratory chain complexes (Desai et al., 2024).

#### 3.3.6 Immune modulation and tumor microenvironment

The tumor microenvironment of PDAC is marked by a dense desmoplastic stroma together with profound immune suppression (Truong and Pauklin, 2021). Several investigators have therefore examined whether statins can reshape the interactions between pancreatic cancer cells, stromal elements, and immune populations within this environment.

Minz et al. treated cultures of murine pancreatic stellate cells (mPSC) and human PDAC cancer-associated fibroblasts (CAFs) with gemcitabine, determining the upregulation of PD-L1 and PD-L2 in these cells (Minz et al., 2023). Then, both basal and immune checkpoint upregulated cultures of mPSC/CAFs were treated with statins: simvastatin and fluvastatin downregulated PD-L1 expression in both basal and gemcitabine-treated mPSC and CAFs. Simvastatin and fluvastatin also downregulated PD-L2 in both basal and gemcitabine-treated CAFs. These experiments were translated *in vivo*: murine pancreatic cancer cells were injected into immunocompetent mice. The mice were divided in 4 groups that received different treatments: methyl cellulose, gemcitabine alone, simvastatin alone, and simvastatin + gemcitabine. The mice were sacrificed on day 20 after tumor cell injection. The tumor tissue in the simvastatin + gemcitabine and simvastatin groups showed higher CD8^+^ T cell infiltration and lower Treg cell infiltration than in the other groups. The weights of the tumors in the simvastatin + gemcitabine group were significantly lower than those in the other groups (Minz et al., 2023).

The increase in CD8^+^ T-cell infiltration reported in some studies is encouraging, but infiltration by itself is not enough to demonstrate meaningful antitumor cytotoxicity. Future work should move beyond cell counts and incorporate functional immune endpoints—such as granzyme B expression, IFN-γ production, and markers of T-cell exhaustion—to determine whether the T-cell influx associated with statin exposure translates into effective tumor-cell killing.

In another study, Ako et al. treated the Raw264.7 and J774A.1 macrophage cell lines with lipophilic statins (simvastatin) where the effect was a reduction of pro-inflammatory cytokines, achieved by the disruption of actin-cytoskeleton by reduction of intracellular protein prenylation (Ako et al., 2022). The conditioning of acinar pancreatic cells with statin-treated macrophages reduced acinar-to-ductal metaplasia (ADM). Furthermore, the oral administration of simvastatin for 3 months to KrasG12D; p48-Cre (KC) mice reduced the occurrence of pancreatic intraepithelial neoplasia (PanIn) lesions (Ako et al., 2022).

Macrophage-related effects likewise remain insufficiently characterized in PDAC. Tumor-associated macrophages are central contributors to disease progression and local immune suppression, and their behavior within the PDAC microenvironment deserves closer study. Although Ako et al. showed a reduction in inflammatory cytokine production, subsequent studies should examine whether statins alter macrophage polarization toward M1 or M2 phenotypes, using markers such as CD86, iNOS, CD206, and Arg1, and should also address the metabolic reprogramming of tumor-associated macrophages within the PDAC setting.

In another study (Shinkawa et al., 2022) with the aim to assess PDAC dependency on niche factors for differentiation, a co-culture of PDAC PDOs and CAFs was generated. Eight PDOs were obtained: 3 well-differentiated (grade 1), 3 moderately differentiated (grade 2), and 2 poorly differentiated (grade 3). Based on transcriptional phenotypes of the PDOs, they were divided into two clusters: cluster 1 – basal-like (containing all Grade 3 and some Grade 2, more aggressive, containing genes with roles in epithelial-to-mesenchymal transition (EMT), inflammation, and proliferation, KRAS and PI3K/Akt/mTOR signaling) and cluster 2 – classical. Each organoid was incubated separately with fetal bovine serum (FBS) medium and niche factors, demonstrating a link between differentiation grade and niche factor dependency: the well-differentiated organoids were more dependent on the niche factors than the poorly differentiated, which developed better in serum medium. Afterward, co-cultures of PDOs and CAFs were generated, resulting in higher CAFs affinity for the well-differentiated PDOs compared with moderately and poorly differentiated ones. Interestingly, grade 2 PDOs demonstrated plasticity in relation to CAFs: they formed ductal structures when placed in co-culture with CAFs and solid structures without CAFs. It was further determined that cluster 2 PDOs were addicted to the Wnt pathway by autocrine Wnt and R-spondin (RSPO). It was also established that CAFs generated R-spondin 3 (RSPO3) to grade 1 PDOs and helped their growth. They then performed separate gemcitabine and simvastatin treatment and discovered that more niche-dependent organoids had the mevalonate pathway upregulated and had a better response to simvastatin, and that the poorly differentiated, less niche-dependent PDOs had a lower response to simvastatin and better response to gemcitabine (Shinkawa et al., 2022). This finding has important implications for patient stratification, suggesting that transcriptomic subtyping and mevalonate pathway activity assessment could guide the selection of patients most likely to benefit from statin therapy.

#### 3.3.7 Epithelial-to-mesenchymal transition and tumor plasticity

Statins have complex and sometimes seemingly contradictory effects on epithelial-to-mesenchymal transition (EMT) in PDAC cells. EMT is not a binary switch but rather a spectrum of intermediate states with distinct functional consequences for invasion, dissemination, and colonization capacity. Fluvastatin promoted EMT and reduced the ability of PDAC cells to organize themselves in spheric tridimensional structures (Dorsch et al., 2021). While this may enhance initial cellular dissemination, it simultaneously reduces cell plasticity—the ability to undergo mesenchymal-to-epithelial transition (MET) at distant sites—which is required for metastatic colonization and outgrowth. On the other hand, however, Miao et al. showed that rosuvastatin inhibits EMT by calcium-mediated endoplasmic reticulum stress, thus diminishing PDAC cell metastatic capacity (Miao et al., 2025). These findings suggest that the net effect on metastasis likely depends on the specific statin, dose, and tumor subtype.

In another study, Li et al. built a large organoid biobank – 260 PDOs on which they performed a multi-omics profile and discovered new coding and noncoding potential driver mutations. They discovered that chemotherapy-resistant organoids were high in protein glycosylation and mevalonate pathway activity, which also correlated with EMT. Statins inhibited chemoresistant organoids, suppressed EMT, and had a synergistic action with chemotherapy (Li et al., 2025).

#### 3.3.8 Combination therapeutic strategies

Given the promising evidence on statins’ effect on PDAC proliferation, several studies have examined statins in combination with chemotherapeutic agents, antidiabetics, anti-inflammatory drugs, and novel molecular constructs.

Xing et al. demonstrated that the HMGCR expression is negatively correlated with PDAC sensitivity to 5-fluorouracil (5-FU). They demonstrated that the addition of GGPP, a mevalonate pathway product with a role in prenylation, decreases the 5-FU sensitivity and that the association of simvastatin to 5-FU produces a strong synergistic effect in MIA PaCa-2 line (Xing et al., 2024).

Pitavastatin and metformin showed a synergistic cytotoxic effect on PANC-1 and AsPC-1 cell lines by determining G0 cell cycle arrest, upregulated cleaved poly (ADP-ribose) polymerase 1 (PARP-1), cleaved caspase-3, and inhibiting PI3K/mTOR (Chen et al., 2021). These discoveries are supported by another study (Teper et al., 2023) of four-week-old obese KRASG12D mutant male mice that were treated with simvastatin in combination with metformin, which, compared to female mice or mice treated with either simvastatin or metformin, had a lower risk of PanIn-3. The effect is explained by the fact that simvastatin and metformin, in combination only, decrease YAP/TAZ transcriptional activity (Teper et al., 2023).

In a combination study with anti-inflammatory medicine, it was demonstrated that combination treatment with simvastatin, celecoxib, and tipifarnib decreases the PANC-1 cells ability to form spheres (Xu et al., 2021).

In another combination study, with small interfering RNA (siRNA), it was shown that interferon-stimulated gene 15 (ISG15) knockdown in pancreatic cancer stem cells (CSCs) by siRNA results in the stabilization of HMGCR, increasing the cell cholesterol, making ISG15 low CSCs more susceptible to the action of statin. Sun et al. demonstrated a synthetic lethal interaction between ISG15 and statins by delivering hyaluronic acid-coated nanoparticle of ISG15-siRNA + atorvastatin in cell cultures (Sun et al., 2025).

## 4 Discussion

### 4.1 Synthesis of evidence and mechanistic convergence

**FIGURE 2:**
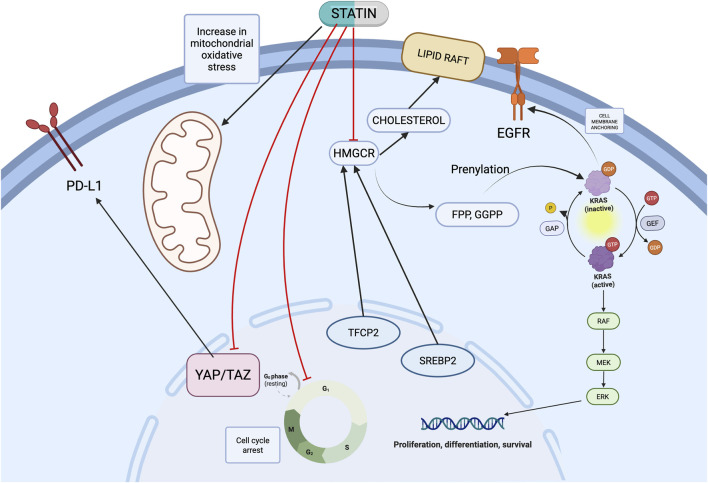
The intracellular mechanisms of action of statins in the PDAC cell (created with biorender.com Accessed on March 2026).

In this scoping review, we have identified four main axes of the antitumoral effect of the statins in PDAC: 1. disruption of various intracellular signaling pathways such as mitogen-activated protein kinases pathway (by blocking the mevalonate pathway which results in lipid rafts disruption and KRAS prenylation) and YAP/TAZ; 2. increase in PDAC cell immunogenicity by downregulating PD-L1 immune checkpoint, increasing intratumoral CD8^+^ T-cell infiltration and decreasing Treg infiltration; 3. microenvironment remodeling: CAF-mediated immune checkpoint expression, macrophage inflammatory cytokine production, and acinar-to-ductal metaplasia; 4. metabolic vulnerabilities: cholesterol depletion by mevalonate pathway blockage, cell cycle arrest, mitochondrial respiration disruption. Intracellular mechanisms of action in the PDAC cell are depicted in Figure 2. These axes should not be viewed in isolation. The mevalonate pathway functions as a central node: its inhibition simultaneously reduces prenylation of KRAS and other small GTPases (disrupting oncogenic signaling), depletes intracellular cholesterol (destabilizing lipid rafts and receptor complexes), and modulates immune-cell behavior (through YAP/TAZ-mediated PD-L1 downregulation). Thus, a single pharmacological event, HMGCR inhibition, propagates across all four mechanistic axes.

### 4.2 Reconciling contradictory clinical evidence

The clinical studies yield mixed results. Preclinical studies are generally consistent in showing antitumor effects. Clinical studies, however, are less uniform, especially for PDAC incidence. The results reported by Okita et al., of a higher PDAC risk after more than 5 years of statin use, may suggest protopathic bias, with early and still unrecognized PDAC causing metabolic changes that require statin treatment. Surveillance bias and confounding by metabolic syndrome are also plausible explanations, since the same factors that lead to statin use may themselves increase PDAC risk. That interpretation is supported by the lack of a similar association in the Hong Kong cohort reported by Xu et al. (2024), as well as by the opposite, apparently protective association observed by Yeh et al. in patients with ILD. These discrepancies suggest that study design and population structure can significantly shape the observed results. Studies of OS have more encouraging results, but they remain constrained by retrospective methodology, limited sample sizes, possible immortal time bias, and incomplete control of confounders. At present, the most important gap in the evidence base is the lack of randomized controlled trial data. Overall, the clinical evidence supporting statin use in PDAC should be considered preliminary: it consists entirely of observational or retrospective analyses and a single uncontrolled phase 2 trial, and clinical validation through prospective randomized studies is still awaited.

### 4.3 Pharmacological considerations

A major translational issue is the difference between the concentrations used experimentally and those that can realistically be reached in patients. *In vitro* work commonly uses statin concentrations between 1 and 50 μM, whereas standard clinical dosing usually produces peak plasma levels in the 10–100 nM range for most statins (Björkhem-Bergman et al., 2011). Notably, the IC50 of statins for their primary target, HMGCR, is in the low nanomolar range (Buccioli et al., 2024), which is well within clinically achievable plasma concentrations. However, the downstream anticancer effects discussed in this review, such as impaired KRAS prenylation, YAP/TAZ downregulation, PD-L1 reduction, and lipid raft disruption, are indirect consequences of mevalonate pathway depletion rather than direct statin–target binding events. For these indirect effects, conventional IC50 values cannot be defined because statins do not directly bind these proteins.

Although mevalonate pathway inhibition is a class effect of the statins, the strength of the effect and its downstream consequences vary by compound, as by the potency hierarchy reported by Uemura et al. Lipophilic statins, including simvastatin, atorvastatin, fluvastatin, lovastatin, and pitavastatin, can cross cell membranes by passive diffusion and may reach higher intracellular concentrations. On the other hand, hydrophilic agents, such as rosuvastatin and pravastatin, depend on transporter-mediated uptake. This pharmacokinetic difference could explain why lipophilic statins have a stronger antiproliferative activity in preclinical systems (Uemura et al., 2023; Rimpelová et al., 2021). Even so, the Norwegian registry analysis by Stoer et al. did not identify a significant OS difference between lipophilic and hydrophilic statins (Stoer et al., 2021).

Drug delivery in PDAC is another challenge. The dense desmoplastic stroma may restrict penetration into the tumor and limit intratumoral exposure. To our knowledge, no study has yet quantified statin concentrations within PDAC tumors, either in patients or in orthotopic models. In that context, newer delivery platforms such as the DZ-SIM nanoconjugate (Ou et al., 2022) and hyaluronic acid-coated nanoparticles (Sun et al., 2025) are of particular interest, because they may help overcome these pharmacokinetic limitations.

### 4.4 Potential biomarkers for statin response in PDAC

The preclinical literature points to several biomarkers that may be useful for selecting patients more likely to benefit from statin treatment. KRAS mutational status is a candidate: Nam et al. reported greater sensitivity in KRAS-mutant compared with wild-type cancers, although this advantage appeared to disappear when BRAF mutations were also present (Nam et al., 2021). Most of the available preclinical studies have been performed in KRAS G12D models, and it remains unclear whether other KRAS variants, such as G12V or G12R, differ in their dependence on prenylation and, by extension, in their susceptibility to statins. HMGCR expression may also be relevant, given the inverse association with 5-FU sensitivity (Xing et al., 2024). Gu et al. identified KIF11, a driver of SREBP2-dependent mevalonate pathway activation, as a predictive biomarker of response to atorvastatin. In addition, Shinkawa et al. observed that niche-dependent, well-differentiated organoids with increased mevalonate pathway activity were the most responsive to statins, raising the possibility that transcriptomic subclassification, for example, classical versus basal-like, could inform patient selection (Shinkawa et al., 2022). Prospective incorporation of such biomarkers into future trials would strengthen the translational relevance of this field. In practice, these biomarkers could be incorporated into future trial designs as stratification factors. For example, in future clinical trials with statins in PDAC, endpoints such as overall survival or progression-free survival could be stratified by KRAS variant or HMGCR expression, potentially allowing the prediction of response to statins based upon these biomarkers.

### 4.5 Knowledge gaps and future research priorities

The lack of clinical trials with statins in PDAC patients is probably the most important gap in the knowledge of the statins’ effect on PDAC. To the best of our knowledge there are currently no phase 3 randomized clinical trials with statins in PDAC and the only phase 2 statin clinical trial in PDAC patients is NCT06241352 (Li et al., 2025). Initiation of statins clinical trials with statins in PDAC, with biomarker stratification (HMGCR expression, KRAS mutation subtype) and in combination with different types of anti-cancer systemic drugs (chemotherapy, targeted therapies such as KRAS inhibitors, immunotherapy) would represent a research priority. Another unexplored research direction is the combination of statins and KRAS inhibitors, especially the emerging pan-KRAS and KRAS G12D inhibitors. The KRAS inhibitors act directly on the KRAS protein and the statins decrease KRAS prenylation, impairing its functionality. Thus, studying whether an additive or synergistic effect of statins and KRAS inhibitors exists is a relevant future research direction. Another important gap in knowledge is represented by the uncertain biological significance of the reported immune-cell infiltration. It should be addressed through studies that include detailed immune phenotyping and functional tests. Another research priority would be the carrying out of pharmacokinetic studies measuring intratumoral statin concentrations in orthotopic models and clinical specimens are needed to establish whether therapeutically relevant concentrations are achievable.

### 4.6 Limitations

The limitations of this scoping review should be noted. This review included only studies from the past 5 years with the aim of analyzing the most recent evidence, however with the risk of excluding older but still relevant studies. Only two databases, PubMed and Web of Science, were searched. Inclusion of other databases might have identified additional studies. Furthermore, because of the data heterogeneity, we presented the findings from the literature in a narrative fashion, in the setting of a scoping review, and could not perform a systematic review with a meta-analysis. The scoping review design does not include formal quality assessment of individual studies. Finally, the predominance of observational designs in the clinical evidence and the use of supraphysiological statin concentrations in many preclinical studies limit the translational conclusions that can be drawn.

## 5 Conclusion

Overall, the evidence gathered in this scoping review supports the idea that statins have antitumoral effects in PDAC through multiple, interconnected mechanisms, such as blocking the mevalonate pathway, impairing oncogenic intracellular signaling, increasing tumoral immunogenicity and remodeling of the tumor microenvironment. The preclinical data are, in several cases, encouraging for combination approaches with chemotherapy. On the other hand, the clinical evidence is still insufficient to support statin use as antitumoral therapy in PDAC, as it is based mainly on observational studies with contradictory findings and a single-arm phase 2 trial. Future research should focus on three priorities: randomized trials with biomarker-guided patient selection, combination studies with KRAS inhibitors, and translational studies incorporating both functional immune analyses and validated predictive biomarkers. Statins remain attractive candidates for drug repurposing in PDAC because they are widely available, inexpensive, and generally well tolerated. Even so, translating preclinical results into clinical practice will require the level of evidence that only carefully designed clinical trials can deliver.

## Data Availability

The original contributions presented in the study are included in the article/supplementary material, further inquiries can be directed to the corresponding author.
